# Obituary: Dr. Laurent Descarries (1939–2012)

**DOI:** 10.3389/fnsyn.2012.00007

**Published:** 2012-12-20

**Authors:** Louis-Eric Trudeau

**Affiliations:** Department of Pharmacology, GRSNC, Université de MontréalMontreal, QC, Canada

**Figure:**
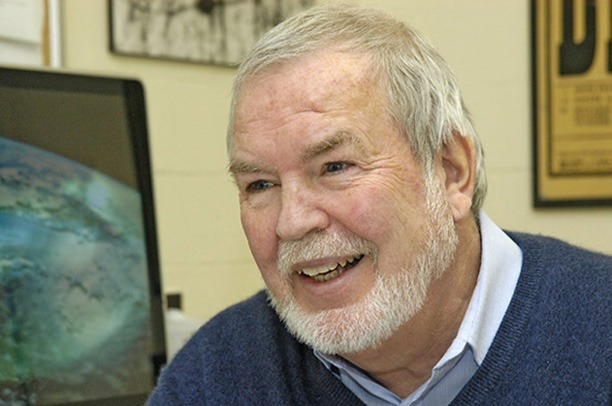
Dr. Laurent Descarries. Credits: Claude Lacasse, Université de Montréal.

On October 3rd 2012, one of Canada's leading neuroscientists, Dr. Laurent Descarries, a professor at the Université de Montréal, passed away at the age of 73. Dr. Descarries was internationally renowned for his extensive work on the ultrastructural organization of monoaminergic and cholinergic innervations in the brain, and in particular for his work demonstrating the mostly asynaptic character of their axon terminals. The latter are now commonly thought to mediate their signals through a process called “diffuse transmission” or “volume transmission.” In the book “*Volume Transmission Revisited*” edited in 2000 by the Swedish neuroanatomist, Kjell Fuxe, Fuxe writes in the introduction: “The theme of our meeting started out more than a century ago with the Nobel laureate Golgi, but after then it disappeared from view until the seventies when, especially Descarries, suggested that central monoamine neurons could communicate with other neurons without recourse to classical synaptic specialisations.”

**Figure 1:**
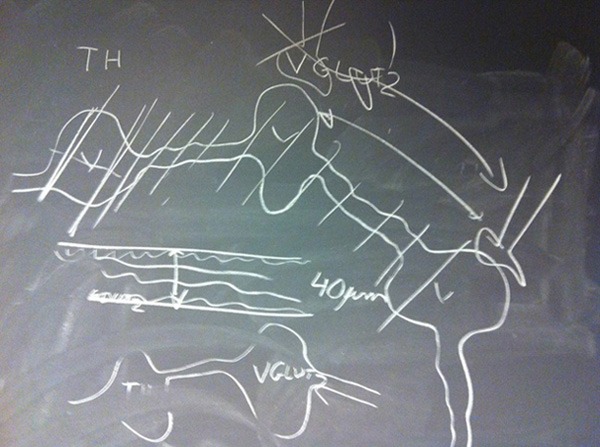
**Schematic diagram hand drawn by Laurent Descarries in December 2011, illustrating the formation of non-synaptic axon terminals by dopamine neurons.** Credit: Åsa Wallén-Mackenzie, Uppsala University.

Trained initially as an MD (Montréal, 1961) and neurologist (Montréal, 1961–1966), Dr. Descarries began his research career during a fellowship at the Massachusetts General Hospital of Harvard University in Boston (1963–1966). During this period, he worked with neurologist Otto Appenzeller on peripheral nervous system dysfunctions associated with cerebrovascular disease. He then pursued his training in neuroanatomy with the renowned anatomist Bernard Droz at the Centre d'Études Nucléaire of Saclay, in France (1967–1969), with whom he contributed to the popularization of the use of autoradiography in combination with electron microscopy to examine the anatomical bases of chemically-defined neurotransmission in the brain.

Laurent Descarries was recruited to the Faculty of Medicine of the Université de Montréal in 1969, where he subsequently remained for his entire career. Funded by a long series of grants obtained from the Medical Research Council of Canada and subsequently from the Canadian Institutes of Health Research, his group published a number of landmark papers on the ultrastructural characterization of noradrenergic, dopaminergic, serotoninergic, and cholinergic axon terminals in the rat and mouse brain, much of which formed the basis of the diffuse transmission theory. This large body of work was published in more than 140 primary research articles and reviews, in addition to many book chapters.

Laurent Descarries was a leading figure of the Canadian neuroscience scene throughout his career. He participated in the establishment of the Centre de Recherche en Sciences Neurologiques (CRSN) at the Faculty of Medicine of the Université de Montréal in 1971, to unite neuroscientists from the University's main campus and affiliated hospitals. He was also one of the founding members of the Groupe de Recherche sur le Système Nerveux Central (GRSNC), a group funded by the Québec government (FRQS) and that currently brings together neuroscientists from the Université de Montréal's main campus. He served as secretary of the Université de Montréal's Faculty of medicine between 2004 and 2006. He was also the main organizer of a number of GRSNC international neuroscience symposia. The outstanding contributions to research of Dr. Descarries were recognized by the Québec research community which selected him as the laureate of the Léo-Pariseau prize for biomedical research in 2005. Laurent Descarries was also recognized as an exceptional mentor: he trained more than 60 graduate students, postdoctoral fellows, and visiting scientists, many of whom now have their own laboratory or are leading figures of the Canadian research scene, including Alain Beaudet, Philippe Séguéla, Patrick Cossette, Mario Beauregard, Guy Doucet, Jean-Jacques Soghomonian, Jean-Paul Soucy, Naguib Mechawar, and Martin Parent.

As colleagues, collaborators, and friends of Laurent Descarries, we salute his outstanding career and scientific contributions and we will miss him dearly.

Louis-Eric Trudeau, Department of Pharmacology, GRSNC, Université de Montréal, Montreal, QC, Canada.

Pierre Drapeau, Department of Pathology and Cell Biology, Université de Montréal, Montreal, QC, Canada.

Trevor Drew, Department of Physiology, Director of the GRSNC, Université de Montréal, Montreal, QC, Canada.

Serge Rossignol, Department of Physiology, Université de Montréal, Montreal, QC, Canada.

Naguib Mechawar, Department of Psychiatry, Douglas Institute, McGill University, Montreal, QC, Canada.

Alain Beaudet, President, Canadian Institutes of Health Research, Ottawa, ON, Canada.

